# Effect of pantoprazole on I-R-induced myocardial injury in diabetic rats targeting inflammatory cytokine release and oxidative stress

**DOI:** 10.22038/ijbms.2021.51624.11714

**Published:** 2021-05

**Authors:** Gaurav Taneja, Arun K. Sharma, Deepa Khanna, Satyendra K. Rajput

**Affiliations:** 1 Department of Pharmacology, Amity Institute of Pharmacy, Amity University, Uttar Pradesh-201303, India; 2 Cardiovascular Division, Department of Pharmacology, Amity University Haryana, Gurugram-122413, India; 3 Rajendra Institute of Technology and Sciences (RITS), Sirsa, Haryana, India; 4 Gurukul Kangri Vishvidyalaya Haridwar, Uttarakhand, India

**Keywords:** Cardiomyopathy, Diabetes mellitus, Inflammatory cytokines, Myocardial I/R insult, Oxidative stress, Pantoprazole

## Abstract

**Objective(s):**

To evaluate the pleiotropic potential and underlying mechanism of pantoprazole (PPZ) (common Proton Pump Inhibitors, PPIs) in type 2 diabetes mellitus (T2DM) -associated ischemia/reperfusion (I-R)-induced myocardial infarction which is still uncharted. Whereas some other PPIs have demonstrated their anti-diabetic, antioxidant, and anti-inflammatory potential.

**Materials and Methods:**

We evaluated the potential of coinciding treatment of PPZ (4 mg/kg/po/day for 8 weeks) in Wistar albino rats against STZ (50 mg/kg/IP) induced T2DM model and I-R provoked cardiac infarction model in diabetic and non-diabetic condition.

**Results:**

PPZ significantly inhibited the perturbed deviations in blood glucose concentration, HbA1c, C-peptide, plasma insulin, and ameliorated the lipid profile (dyslipidemia). PPZ protected myocardial tissue against lipid peroxidation by restoring the levels of serum TBARS and reduced NBT. The significant protective effects of PPZ were evident by ameliorating CKMB, LDH, cTnI, and myocardial oxidative stress in PPZ treated animals. Additionally, PPZ prominently reduced various proinflammatory cytokines release including TGF-β1, TNF-α, and IL-6. PPZ upsurges the bioavailability of nitrite/nitrate concentration which may pacify the impact of myocardial infarction in diabetic I-R injury.

**Conclusion:**

The consequences indicate that PPZ possesses a potent protective effect against diabetic I-R-induced myocardial infarction via suppressing oxidative stress, inflammation, and dyslipidemia-associated tissue damage.

## Introduction

International Diabetes Federation (IDF) facts revealed that the quotient of type 2 diabetes mellitus (T2DM) is progressively rising in the young population as around 352 million people (working population) are suffering from diabetes whereas the anticipated elevation in this statistics is to 417 million by 2030 and to 486 million by 2045 (1). This incessantly increasing prevalence of T2DM directly intensifies the incidence of diabetic vasculopathy including cardiomyopathy by triggering vascular endothelial dysfunction, nitric oxide scavenging, oxidative stress, and platelet aggregation by stimulating the non-enzymatic glycation of surface proteins (2, 3). Moreover, chronic T2DM produces numerous inflammatory cytokines which stimulate the apoptotic/necrosis and inflammatory cascades (2, 4). 

The structural arrangement of proton pump inhibitors (PPIs) comprised a common scaffold of benzimidazolide conjugate (benzimidazole) which is known for the core cavity of diverse therapeutic agents including opioid, antihistaminic, antihelmintic, and anticancer drugs (5). PPIs have been also revealed as ameliorative agents against diverse cardiovascular diseases as they may lead to vascular relaxation (6), reduce atrial fibrillation (7), and have positive inotropic and negative chronotropic consequences (8). Recent investigation revealed the substantial anticancer effect of omeprazole in human colon cancer cell lines (9). Moreover, the treatment of omeprazole and lansoprazole has also been supported for diminishing the occurrence of tuberculosis (10). Pantoprazole (PPZ) is a conventionally used PPIs for the prevention of gastroesophageal reflux disease and hypersecretory gastric disorders including Zollinger-Ellison syndrome (11). Beyond its anti-ulcer effects, PPZ has been investigated for its pleiotropic effects. Recent reports revealed significant therapeutic potential of PPZ including anti-inflammatory (12), anti-proliferative (13), antiapoptotic and antioxidant properties (14). PPZ has been studied for its protective potential against ischemia/reperfusion (I-R) induced myocardial infarction where infarct size was significantly lower in the pantoprazole group (15). However, direct evidence of the therapeutic efficacy of PPZ with supportive underlying mechanism against diabetes mellitus is not yet known. The current study was intended to explore the effect of PPZ against diabetic induced cardiomyopathy, I-R induced myocardial infarction and diabetic I-R induced myocardial insult in rats. 

## Materials and Methods

Wistar albino rats of either sex were sanctioned for the present study by the Committee for the Purpose of Control and Supervision of Experiments on Animals (CPCSEA), New Delhi, India (approval No: RITS/IAEC/2016/07/07). All animals were first acclimatized for one week with allowance of *ad libitum* rat chow and drinking water*. *The animals were accommodated with the usual cycle of day-and-night, humidity of 60% ± 10%, and at 25 ± 5 °C temperature.

***Design of experiment***

**Figure 1:**
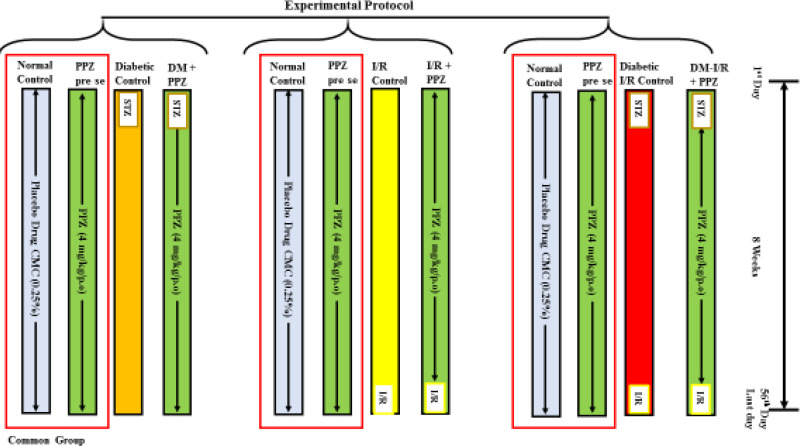
Notion represents the summary of the experimental protocol with details of treatments

A total of 48 animals were classified into 8 groups where each group had six animals (n=6, about 200–220 g), were used in the current study, namely normal control, diabetic control, I-R control, diabetic I-R control, drug *per se*, and three PPZ treated groups against diabetic control, I-R control, diabetic I-R control. PPZ at a dose of 4 mg/kg/*po* suspended in 0.25% carboxymethylcellulose was administered daily for 8 weeks. The summary of the experimental protocol is given in Figure 1: 

Group I (Normal Control), rats were accommodated with allowance of *ad libitum* rat chow and drinking water. Carboxymethylcellulose (0.25%) was given to rats as a placebo (8 weeks). 

Group II (Diabetic Control), rats received a single dose of STZ (50 mg/kg/IP, once) dissolved in freshly prepared 0.1 M citrate buffer (pH 4.5) to develop the T2DM model. 

Group III (I-R control), rats were exposed to 45/60 min I-R injury on the last day to develop MI. 

Group IV (Diabetic I-R control); animals received STZ (50 mg/kg/IP, once) on the first day to developed the T2DM model, and on the last day of study, animals were exposed to cardiac insult by I-R injury. 

Group V (Drug per se) animal had regular treatment with PPZ (4 mg/kg/PO) for 8 weeks. 

Group VI (PPZ in T2DM), diabetic rats (as detailed in group II) were administered a regular treatment with PPZ (4 mg/kg/*po*) for 8 weeks after confirming the induction of diabetes. 

Group VII (PPZ in I-R), animals were treated with the regular dose of PPZ for 8 weeks followed by I-R exposure on the last day as detail in group III. 

Group VIII (PPZ in diabetic I-R), diabetic animals received daily treatment with PPZ (4 mg/kg/*po*) for 8 weeks followed by I-R exposure as detail in group IV.

***Induction of T2DM model and cardiac-toxicity***

STZ (Himedia Laboratories Pvt. Ltd, Mumbai, India) was used to induce experimental T2DM. STZ solution was prepared by mixing the dry powder of STZ in fresh citrate buffer (0.1 M cold, pH 4.5). A single dose of freshly prepared STZ (50 mg/kg/day/once) solution was administered to the overnight fasted rats of either sex (weight: 200–220 g; age group: 6–7 weeks) by intraperitoneal route. To confirm the presence of diabetes in a rat model, the level of blood glucose was evaluated afterward to 72 hr of STZ injection. Only animals having blood glucose levels more than 300 mg/dl were considered diabetic. The diabetic rats were further studied after 8 weeks to evaluate the development of cardiomyopathy.

***Induction of experimental I-R injury***

Animals submitted to the different treatments were further exposed to I-R injury to measure the cardiac remodeling. Initially, the animals were injected with ketamine (90 mg/kg) and xylazine (10 mg/kg) intraperitoneally to induce anesthesia. For inducing I-R injury a little cut was made on the neck and the trachea was identified. The tube of rodent respirator (60 breaths/min) was introduced into the trachea and secured with 6-0 silk suture. A “knot anchor” was created and used to secure the cannula to the trachea. Anesthetized rats were subjected to left thoracotomy, and the left anterior descending coronary artery was tied by surgical stitch (6-0) for 45 min followed by 60 min reperfusion (16). 

***Method for serum sample collection, biochemical and histological evaluation***

All animals got abstained from food overnight and then euthanized by administering a higher dose of anesthesia (ketamine 90 mg/kg and xylazine 10 mg/kg) on termination day of the experimental protocol. The blood sample was collected through retro-orbital plexus puncture and permitted to clot before centrifugation (10,000 g for 10 min at 2–4 °C). The subsequent supernatant was cautiously collected as serum and further consumed for assessment of biochemical parameters (cTnI, CK-MB, LDH) using commercially available kits. The serum nitrite/nitrate ratio was estimated as described below. 

***Estimation of diabetic and myocardial biochemical parameters***

The measurement of T2DM was estimated by blood glucose level (glucometers, Accu Chek Active Blood Glucose Meter Kit). In addition, glycated hemoglobin A1c (HbA1c) level, rat insulin level, and rat C-peptide level were estimated by ELISA kits, available in the market. HbA1c ELISA kit was obtained from Genxbio Health Sciences Pvt. Ltd. Delhi, India, rat C-peptide ELISA kit and rat insulin ELISA kit were procured from RayBiotech, Norcross, GA, USA. Lipid profile including total cholesterol (TC), high-density lipoprotein (HDL), low-density lipoprotein (LDL), and triglycerides (TG) was assessed by commercially available kits (procured from Erba Diagnostics, Inc, USA). Diabetes or I-R -induce cardiac insult was assessed by CKMB, LDH, and cTnI levels in serum using commercially available kits. Estimation of cardiac biomarker including LDH, CKMB, and cTnI was done by commercially available enzymatic kits which were procured from Transasia Bio-Medicals Ltd, India and Logotech India Pvt Ltd, India, respectively. 

***Estimation of serum nitrite/ nitrate concentration***

The bioavailability of nitric oxide was estimated by measuring the serum nitrite/nitrate concentration as conferred in our previous study (3, 16). “In brief, to measure the serum nitrite/nitrate concentration a mixture of carbonate buffer (400 μl) of pH 9.0 and serum sample (100 μl) was prepared by presence of small amount (~0.15 g) of copper-cadmium alloy and stored for 1 hr at room temperature. Then 100 μl of NaOH was mixed in the solution to stop the reaction. The serum content of the mixture was deproteinized by adding a ZnSO_4 _solution for about 10 min and then centrifuging the mixture. Greiss reagent was included in the supernatant, and absorbance was measured at 545 nm” (17). Moreover, the evaluation of proinflammatory cytokines (TNF-α, IL-6, and TGF-β) was determined by enzyme-linked immunosorbent assay (sandwiched ELISA kit, Ray Biotech, USA) using serum sample (as per manual of manufacturer’s protocol). 

***Method of tissue collection and histological evaluation***

After exsanguinations, the heart and pancreatic tissues were excised from each animal and preserved in a nitrogen container. The lower part of the myocardial tissue (ventricle) was minced and mixed with phosphate-buffer (5 ml) and centrifugated at 10,000 g for 15 min. This homogenized cardiac sample was used to estimate TBARS, superoxide anion generation (measured by reduced NBT level) in cardiac tissue. Whereas, for histological assessment, the isolated heart and pancreatic tissues were collected in 5% neutral formalin solution and planted in paraffin wax using the tissue-embedding center. The small sections were cut down using microtome and specimens were marked with hematoxylin-eosin staining. The tainted specimens were spotted using an optical microscope (3).

***Estimation of serum and myocardial oxidative stress***

The oxidative stress in serum and tissue samples was calculated by estimating MDA level using thiobarbituric acid reactive substances (TBARS) and superoxide anion generation measured by reduced NBT level, respectively. 

***Statistical analysis***

All data points were stated in mean±SD form and statistical analyses were examined by one-way ANOVA and Tukey’s multiple comparison test (*post hoc*) using SigmaPlot (version 12.0) maintained by SYSTAT Software, Inc (USA). The exact value of *P* has been given in parenthesis. A result having a *P*-value below 0.05 was counted as statistically significant.

## Results

***Effect of PPZ on hyperglycemia***

**Figure 2:**
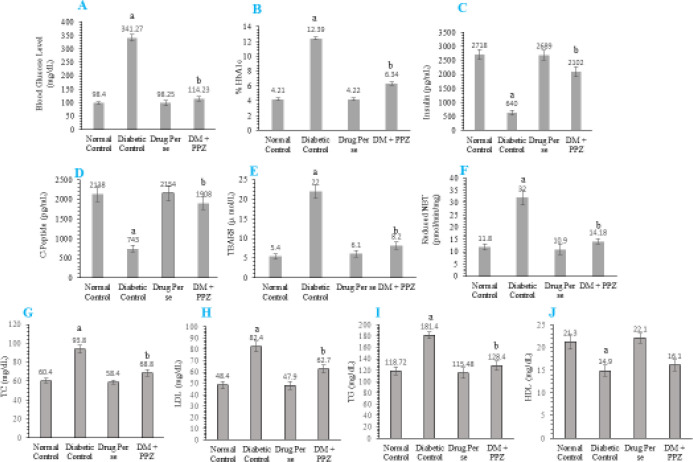
Graphical notation represents the effect of PPZ on blood glucose level (mg/dl) (A), %HbA1c (B), insulin (pg/dl) (C), C-peptide (mg/dl) (D), serum TBARS (µ mol/l) (E), reduced NBT level (pmol/min/mg) (F) and lipid profile inkling TC (mg/dl) (G), LDL (mg/dl) (H), TG (mg/dl) (I), and HDL (mg/dl) (J). All values were stated as mean±SD where facts were examined by one-way ANOVA and *post hoc* Tukey’s multiple comparisons test, ‘a’ denotes *P*<0.001 in comparison with normal; ‘b’ denotes *P*<0.001 in comparison with the diabetic group

PPZ: Pantoprazole; HbA1c: hemoglobin A1c; TBARS: thiobarbituric acid reactive substances; TC: total cholesterol; LDL: low-density lipoprotein; TG: triglycerides; HDL: high-density lipoprotein

The occurrence of T2DM due to administration of STZ (50 mg/kg/IP) in normal rats was evaluated by increased level of blood glucose (mg/dl) and % HbA1c, and reduced level of insulin (pg/dl) and C-peptide (pg/dl). In addition, the increased oxidative stress in diabetic animals was further analyzed by measuring the increased level of serum TBARS (µ mol/l) and reduced NBT level (pmol/min/mg). T2DM induced dyslipidaemia were studied by increased level of TC (mg/dl), LDL (mg/dl), TG (mg/dl), and slightly reduced level of HDL (mg/dl) in comparison with normal control animals. Treatment with PPZ in diabetic animals significantly reduced the pathological alteration. PPZ treated diabetic animals showed a significantly reduced level of blood glucose and % HbA1c, and upsurged level of insulin and C-peptide in comparison with the T2DM control group. Additionally, the treatment with PPZ in T2DM animal model for measurement of reactive oxygen species (ROS) showed a significant reduction of serum TBARS and reduced NBT in comparison with the T2DM control group. Moreover, the treatment with PPZ significantly overcame the increased level of TC, LDL, and TG in T2DM animals in comparison with the T2DM control group. Whereas PPZ treatment showed a negligible upsurge of HDL level as compared with diabetic control animals (Figure 2). 

***Effect of PPZ on cardiomyopathy and I-R induced myocardial infarction in diabetic and non-diabetic rats***

**Figure 3:**
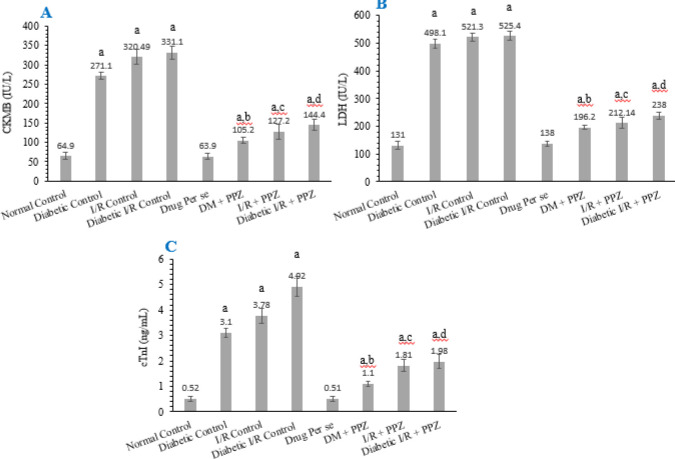
Figure describes the effect of PPZ on CKMB (IU/l) (A), LDH (IU/l) (B), and cTnI (ng/ml) (C). All values were stated as mean±SD where facts were examined by one-way ANOVA and *post hoc* Tukey’s multiple comparisons test, ‘a’ denotes *P*<0.001 in comparison with normal; 'b' denotes *P*<0.001 in comparison with the diabetic group; ‘c’ denotes *P*<0.001 in comparison with I-R control and ‘d’ denotes *P*<0.001 in comparison with diabetic I-R control

PPZ: Pantoprazole; HDL: high-density lipoprotein

Myocardial tissue was exposed to different pathological conditions and significant injury was observed, measured by increased level of CKMB (IU/l) in the respective pathological state including T2DM induced cardiomyopathy, I-R -induce MI, and diabetic I-R induced myocardial damage. Significantly increased levels of LDH (IU/l) were observed in the respective pathological state including T2DM induced cardiomyopathy, I-R -induced MI, and diabetic I-R induced myocardial damage and similarly, a significant increase in cTnI (ng/ml) were observed in all pathological states including T2DM induced cardiomyopathy, I-R -induced MI, and diabetic I-R induced myocardial damage in comparison with the normal control group. More specifically, the increased level of these biomarkers was comparatively higher in the diabetic I-R control group which indicates the presence of severe injury in myocardial tissue. Whereas the treatment with PPZ against all three pathological conditions (T2DM control, I-R control, and diabetic I-R control) exhibited substantial diminution in CKMB level, LDH, and cTnI level against each pathological condition, respectively in comparison with their respective diseases control groups (Figure 3). 

***Effect of PPZ on inflammatory cytokines***

**Figure 4:**
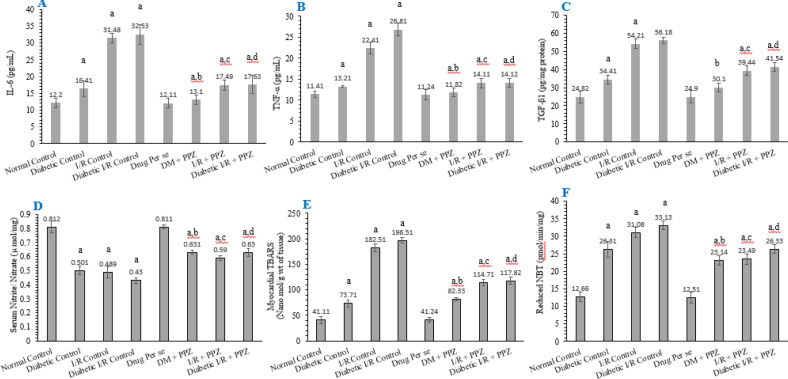
Figure depicts the effect of PPZ on IL-6 (pg/ml) (A), TNF-α (pg/ml) (B), TGF-β1 (pg/mg protein) (C), serum nitrite/nitrate concentration (µ mol/mg) (D), myocardial TBARS (nano mol/g wt of tissue) (E) and myocardial reduced NBT (pmol/min/mg) (F). All values were stated as mean±SD where facts were examined by one-way ANOVA and *post hoc* Tukey’s multiple comparisons test, ‘a’ denotes *P*<0.001 in comparison with normal; 'b' denotes *P*<0.001 in comparison with the diabetic group; ‘c’ denotes *P*<0.001 in comparison with I-R control and ‘d’ denotes *P*<0.001 in comparison with diabetic I-R control

PPZ: Pantoprazole; TBARS: thiobarbituric acid reactive substances

The exposure to myocardial I-R in diabetic rats substantially raised the production of cytokines in comparison with the normal control. The present outcomes revealed the increased level of IL-6 (pg/ml), TNF-α (pg/ml)*, *and TGF-β1 (pg/mg protein) in diabetic, I-R, and diabetic I-R treated animals, respectively. Conversely, treatment with PPZ in T2DM animals significantly reduced the serum level of IL-6, TNF-α, and TGF-β. In addition, treatment with PPZ also showed similar consequences in I-R and diabetic I-R exposed animals for IL-6, TNF-α, and TGF-β (Figure 4).

***Effect of PPZ on serum nitrite/ nitrate concentration and myocardial oxidative stress***

The level of serum nitrite/nitrate concentration (µ mol/mg) was significantly reduced in the diabetic group, I-R -induced MI group, and diabetic I-R induced myocardial damage group in comparison with a group of normal control animals. Whereas treatment with PPZ against these disease groups significantly increased the level of serum nitrite/nitrate concentration including T2DM animal model, I-R -induced MI rats, and diabetic I-R induced myocardial damage rats in comparison with their respective diseases control group (Figure 4). 

In addition to all specific biomarkers the myocardial oxidative stress was measured. Significant elevation of myocardial oxidative stress (accessed by myocardial TBARS (Nano mol/g wt of tissue) and reduced NBT level (pmol/min/mg), respectively) were calculated in diabetic animals, I-R exposed rats, and diabetic I-R insulted rats in comparison with normal control rats. The reduced level of myocardial TBARS and reduced NBT level was measured in PPZ treated animals *vs* diabetic control, *vs* I-R control, and *vs* diabetic I-R control group.

***Effect of PPZ on histological alteration in pancreatic and cardiac tissue***

**Figure 5:**
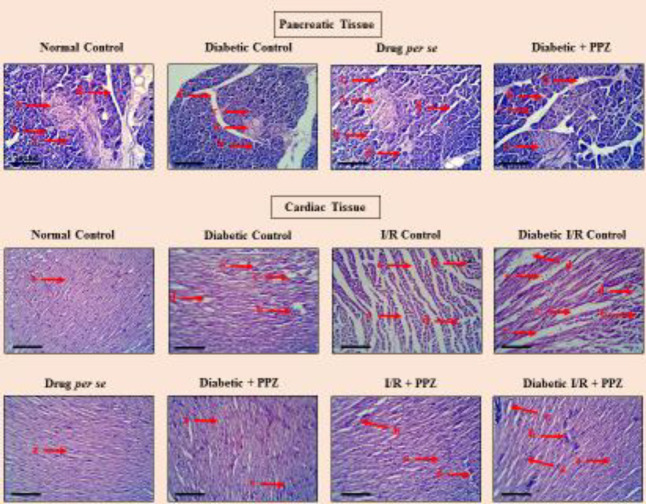
Figure portrays the effect of PPZ on the histological examination of pancreatic and cardiac tissues using an inverted microscope (Cosmo Laboratory Equipment) at 40X (scale bar=100 μm). The histological architecture of pancreatic tissue was categorized as 'a' characterizes a normal circular shape of islets of Langerhans, 'b' characterizes acinar cells, 'c' characterizes pancreatic lobules, and 'd' represents interlobular connective tissue septa. Histological examination of myocardial tissue was also categorized as 'a' characterizes normal myocardial fibers, 'b' characterizes contraction band necrosis, 'c' characterizes perivascular cuffing, 'd' characterizes increased intercalated space, and 'e' characterizes nuclei karyolysis

The histological valuation of pancreatic tissue revealed no structural lesions in the normal control and drug *per se* group. The histological valuation of T2DM control rats showed significant structural impairments including squeezed or damaged islets of Langerhans, swollen acinar cells, and flattened interlobular duct in comparison with the normal control group. The histological observation of PPZ treated animals showed mild damage in the islets of Langerhans cells and interlobular duct as well as no swelling in acinar cells (Figure 5). Scoring of structural alteration in the histological images of pancreatic muscle was cited in Table 1. 

Moreover, the histology of myocardial tissue of the normal control group showed no sign of cytoplasmic vacuolization or myofibrillar loss. Whereas, a significant presence of perivascular cuffing, band necrosis, nuclei karyolysis, and increased intercalated space (widened intracellular space) were observed in diabetic control, I-R control, and diabetic I-R control groups. Intriguingly, it was discerned that the impact of cardiac damage was significantly more in the diabetic I-R control group in comparison with the individual influence of T2DM and I-R induced cardiac damage. PPZ significantly reduces the consequences of pathological conditions by reducing perivascular cuffing, band necrosis and intercalated space against diabetes-induced cardiomyopathy, I-R and diabetic I-R induced myocardial infarction (Figure 5). Scoring of structural alteration in the histological images of myocardial muscle was cited in Table 2. 

## Discussion

PPZ is a commonly used PPI against gastroesophageal reflux disease and other gastric disorders (18)K-ATPase in order to inhibit gastric acid secretion. Omeprazole, lansoprazole, pantoprazole, rabeprazole and esomeprazole belong to PPIs category. Although PPIs have a good safety profile, allergic reactions to these molecules can occur. The real rate of hypersensitive reactions to PPIs is unknown. The aim of this retrospective study is to evaluate the rate of hypersensitive reactions to PPIs in patients admitted to our Unit between 2008 and 2013 with a history of drug hypersensitivity. From a database of 1229 patients (921 women, 308 men. However, the recent investigations accentuate the pleiotropic actions of PPI (PPZ), including anti-inflammatory (12), anti-proliferative (13), antiapoptotic and antioxidant properties (14). The consequences of some recent clinical studies showed a lower risk of hyperglycemia by PPIs treatment (19, 20). Indeed, the direct evidence with supported underlying mechanism for therapeutic potential of PPZ against T2DM and induced cardiomyopathy and especially diabetic I-R are still unknown. Moreover, the structural scaffold (multitudinous possibility of interaction with biological receptors), broad safety range, and pleiotropic therapeutic effect (as suggested by recent reports) of PPZ develop a need for a revisit to explore its concealed therapeutic potential. Our study indorses the severe consequences where a significant increase of serum TBARS, reduced NBT level, and perturbed level of lipid profile (increased level of TC, LDL, TG, and reduced level of HDL) was observed in diabetic animals as reported by recent investigations (21, 22). Whereas the treatment with PPZ significantly blocks the continuous surge of blood glucose, HbA1c, TBARS, reduced NBT level, and lipoprotein concentration (TC, LDL, and TG) in diabetic animals. The anti-hyperglycaemic effect of PPZ was further measured by the upsurge level of insulin, C-peptide, and HDL. The significant changes in the mentioned biomarkers substantiate the anti-diabetic effect of therapeutic intervention as reported in recent studies (23). The increased level of specific cardiac biomarkers including CKMB, LDH, and cTnI, and reduced level of serum nitrite/nitrate concentration indorses the existence of myocardial injury in diabetic and I-R control groups, either individually or in combination. Also, the increased level of IL-6, TNF-α, and TGF-β1 showed the induction of pro-inflammatory cytokines in all three pathological conditions which revealed that chronic T2DM and I-R insult significantly up-regulate the release of inflammatory cytokines as reported previously (3, 16). Yu *et al*. 2011 confirmed the association of hyperglycemia-induced release of proinflammatory cytokines in micro and macrovascular complications (4). Moreover, the increased level of myocardial oxidative stress also performs a prominent part in the induction of cardiac damage as free-radical generation sparks to cell membrane peroxidation and distraction of cardiac myocytes (24). A similar observation has been found in our study which revealed that diabetic and associated I-R can cause severe myocardial damage. Treatment with PPZ significantly regulated the perturbed level of biomarkers which suggests the cardioprotective potential of PPZ against diabetes, I-R, and diabetic I-R injury. 

The diminished level of inflammatory cytokines in diabetes, I-R and diabetic I-R treated animals supports the anti-inflammatory potential, and diminished level of oxidative stress in these pathological groups indicates the antioxidant effect of PPZ. Whereas, the upsurged nitrite/nitrate concentration level by PPZ in T2DM, I-R and diabetic I-R treated animals divulged its potential to improve the bioavailability of nitric oxide, which is the prime factor of endothelium having the ability to protect myocardial blood vessels during myocardial infarction (25). In addition, the histological evaluation in different groups of animals with their respective treatment endorses the structural changes. DM-induced β-cells destruction was observed by squeezed or damaged islets of Langerhans, swollen acinar cells, and flattened interlobular duct. These consequences were supported by other published reports (26–28). Whereas treatment with PPZ significantly reverts the pathological changes which indicate the anti-inflammatory and antioxidant potential of PPZ. Moreover, the association of cardiovascular damage was also observed in the histological assessment as represented in previous reports (29, 30). The myocardial structural alteration in the disease group was measured by existence of increased intercalated space, band necrosis, nuclei karyolysis, and perivascular cuffing in T2DM control, I-R control, and diabetic I-R control groups which evidence the initiation of cardiac damage (3, 23). Conversely, comparably to the biomarker’s regulation, treatment with PPZ significantly reinstated the structural indiscretion in cardiac tissue against pathology and confirmed the cardioprotective effect of PPZ. The present investigation also highlights the repurposing of PPZ. PPZ is a widely used PPIs in regular clinical practice, and outpatient settings for short-range therapy of oesophageal acidity. We suggest that despite the therapeutic effects on the GIT system, PPZ also has favorable pleiotropic action on myocardial tissue.

**Table 1:** Scoring of structural alteration in the histological images of pancreatic muscle

Group *vs* structural observation	Squeezed islets of Langerhans	Swollen acinar **c**ells	Pancreatic lobules	Flattened interlobular duct
Normal control	**-**	**-**	**-**	**-**
Diabetic control	**+ + +**	**+ + +**	**+ + +**	**+ + +**
Drug *per se*	**-**	**-**	**-**	**-**
Diabetic + PPZ	**+**	**+**	**+**	**+**

Scoring of structural alteration in the histological images of pancreatic muscle. The alterations are graded as "+++" represents chronic injury, "++" represents moderate injury, "+" represents minor injury, and "−" represents no injury

**Table 2:** Scoring of structural alteration in the histological images of myocardial muscle

Group *vs* structural observation	Normal myocardial fibers	Contraction band necrosis	Perivascular cuffing	Intercalated space	Nuclei karyolysis
Normal control	**+ + +**	**-**	**-**	**-**	**-**
Diabetic control	**-**	**+ +**	**+ +**	**+ +**	**+ +**
I-R control	**-**	**+ +**	**+ +**	**+ +**	**+ +**
Diabetic I-R control	**-**	**+ + +**	**+ + +**	**+ + +**	**+ + +**
Drug *per se*	**+ + +**	**-**	**-**	**-**	**-**
Diabetic + PPZ	**+**	**-**	**-**	**-**	**+**
I-R + PPZ	**+**	**+**	**-**	**+**	**-**
Diabetic I-R + PPZ	**+**	**+**	**+**	**-**	**+**

Scoring of structural alteration in the histological images of myocardial muscle. The alterations are graded as "+++" represents chronic injury, "++" represents moderate injury, "+" represents minor injury, and "−" represents no injury

PPZ: Pantoprazole

## Conclusion

The consequences of the present investigation revealed the anti-diabetic and cardioprotective potential of PPZ against diabetes and I-R injury, which are probably linked to its antioxidant and anti-inflammatory potential. Additionally, PPZ treatment also showed significant improvement against dyslipidemia and bioavailability of nitric oxide. Thus, the consequences indicate that PPZ possesses a potent protective effect against diabetic I-R-induced myocardial infarction via suppressing oxidative stress, inflammation, and dyslipidemia-associated tissue damage.
